# The Worm Turns (and Squirms)

**Published:** 1910-04

**Authors:** 


THE WORM TURNS (AND SQUIRMS).—The protean editor-secretary of the Octopus makes a labored, feeble and altogether futile attempt at defense in the *J. A. M. A.* March 5, and says that Lydston *et al.* "go back 38 years" to rake up his record. This allegation is easily refuted. It has been just eighteen years since he *procured* a diploma from Rush, and twenty-two years since he was running the Lincoln Medical Institute and water cure establishment and advertising as all sorts of a specialist, in the Nebraska newspapers. He says that those famous resolutions by the Chicago Medical Society have been rescinded; but fails to say that it was done in the absence of six members who had voted for the resolutions, and carried by one vote. Quackery, "wounded, writhes in pain and dies amidst its worshipers," or words to that effect.

